# Efficacy and safety of bevacizumab-based maintenance therapy in metastatic colorectal cancer: A meta-analysis

**DOI:** 10.1097/MD.0000000000018227

**Published:** 2019-12-16

**Authors:** Hongbo Ma, Xiaoli Wu, Miaomiao Tao, Nan Tang, Yanyan Li, Xianquan Zhang, Qi Zhou

**Affiliations:** a The Fuling Center Hospital of Chongqing City; b The Second Affiliated Hospital of Chongqing Medical University, Chongqing, China.

**Keywords:** bevacizumab, maintenance therapy, meta-analysis, metastatic colorectal cancer

## Abstract

**Objective:**

To identify the optimal treatment strategy after first-line induction chemotherapy for metastatic colorectal cancer (mCRC).

**Methods:**

We conducted a meta-analysis of randomized controlled trials comparing bevacizumab-based maintenance therapy, observation, and continuous chemotherapy.

We searched the PubMed, Embase, and Cochrane databases for relevant articles published through March 2018. All randomized phase-III trials evaluating bevacizumab-based maintenance treatment after bevacizumab-based induction treatment were eligible for inclusion. The primary and secondary endpoints were progression-free survival (PFS) and overall survival (OS), respectively. Hazard ratios (HRs) with 95% confidence intervals (CIs) or data for calculating HRs with 95% CIs were extracted. The RevMan v5.3 (Copenhagen, Denmark) software was used for data analysis.

**Results:**

Nine trials (3121 patients) were included in this meta-analysis. Compared with observation alone, bevacizumab-based maintenance therapy significantly improved PFS (HR: 0.62, 95% CI: 0.47–0.82) and showed a trend toward prolonged OS (HR: 0.93, 95% CI: 0.83–1.05). The incidence of grade 3/4 toxicity, including hypertension and fatigue, was higher after maintenance therapy than after observation alone. PFS (HR: 0.91, 95% CI: 0.70–1.18) and OS (HR: 0.88, 95% CI: 0.74–1.04) did not differ between the maintenance treatment and continuous chemotherapy groups. Grade 3/4 toxicity, including diarrhea and sensory neuropathy, was less common after maintenance therapy than after continuous chemotherapy.

**Conclusion:**

Bevacizumab-based maintenance therapy significantly improved PFS, showed a trend toward prolonged OS, and reduced cumulative grade 3/4 toxicity relative to continuous chemotherapy with comparable efficacy. Although maintenance therapy was beneficial, the optimal strategy should be individualized.

## 1 Introduction

Colorectal cancer (CRC) is one of the most commonly diagnosed malignancies. In 2012, there were an estimated 1.36 million new cases of CRC and 694,000 CRC-related deaths worldwide.^[1]^ Although the 5-year survival rate of CRC patients has increased from 51% to 65%, and more patients are being diagnosed at earlier stages, about half of all CRC patients will eventually develop metastasis, leading to inoperable metastatic colorectal cancer (mCRC).^[2]^ Moreover, approximately a quarter of all CRC patients present with mCRC at diagnosis.^[3]^ Chemotherapy is the preferred treatment for mCRC patients for whom complete resection cannot be achieved. Over the past few decades, significant advances have been made in mCRC treatment, resulting in improved outcomes and prolonged survival. Several drugs have been developed to treat mCRC, such as oxaliplatin,^[4]^ the fluoropyrimidines 5-fluorouracil (5-FU)^[5]^ and capecitabine,^[6]^ irinotecan,^[7]^ the epidermal growth factor receptor antibodies cetuximab^[8]^ and erlotinib,^[9]^ and the vascular endothelial growth factor (VEGF) antibody bevacizumab.^[10]^ First-line therapy with bevacizumab combined with multi-drug chemotherapeutic regimens (e.g., FOLFOX, XELOX/CAPOX, and FOLFIRI) has increased response rates to 50% to 60%, median progression-free survival (PFS) to 9 to 11 months, and median overall survival (OS) to 30 months in patients with unresectable mCRC.^[11]^

However, there is no consensus on the optimal follow-up treatment strategy—maintenance therapy, continuous chemotherapy, or observation alone—for mCRC patients who benefit from first-line therapy. Continuous chemotherapy leads to an increase in drug-related side effects, and long-term exposure to chemotherapeutic drugs reduces cancer cell sensitivity to drugs, resulting in drug resistance. Moreover, treatment interruption significantly reduces the efficacy of chemotherapy and may even affect a patient's PFS and OS. The concept of maintenance treatment envisages a period of high-intensity chemotherapy, after which those agents that are mainly responsible for cumulative toxicity are stopped. The results from 2 large, prospective, observational studies suggest that continued VEGF inhibition with bevacizumab beyond the initial disease progression could play an important role in improving the overall success of therapy in mCRC patients.^[12,13]^ A comparative assessment of bevacizumab-based maintenance strategies, continuous chemotherapy, and observation alone may help identify the optimal chemotherapeutic regimen for the sequential treatment of mCRC patients who benefit from first-line therapy. We therefore conducted a meta-analysis of randomized controlled trials evaluating the safety and efficacy of the above 3 strategies in terms of PFS and OS in order to identify the optimal follow-up treatment strategy for mCRC patients.

## 2 Materials and methods

### 2.1 Data sources and search strategy

Potentially relevant studies were independently identified by 2 authors who conducted a structured literature search of the PubMed, Embase, and Cochrane Library databases and the meeting abstracts of American Society of Clinical Oncology and European Society for Medical Oncology published through March 2018. The searches were systematically performed using Medical Subject Headings, and the full-text search terms for the literature search included “colorectal cancer,” “bevacizumab,” and “maintenance.” The online abstracts of the retrieved studies were screened for eligibility. The references of all eligible studies were manually reviewed to find additional relevant studies.

### 2.2 Study selection

The inclusion criteria for the studies were as follows: phase III randomized controlled trials involving patients with histopathologically confirmed CRC; studies comparing bevacizumab-based maintenance therapy with observation alone or those comparing bevacizumab-based maintenance therapy with continuous chemotherapy; studies that reported one or more of the primary or secondary outcomes; and studies from which we could directly obtain or calculate hazard ratios (HRs) and 95% confidence intervals (95% CIs).

The exclusion criteria were as follows: studies that had only a single treatment arm; studies in which data on the primary or secondary outcomes were not available; studies for which we were unable to obtain the full text or those that provided insufficient data; and case reports, meeting abstracts, literature reviews, and animal experiments.

### 2.3 Data extraction

Data extraction was performed independently by 2 authors, and the extracted data were entered into a standard data sheet. Data on the following variables were extracted: first author's name, year of publication (acronym of the trial), journal, affiliated institution, country, study phase, format (full text or abstract), interventional and control treatments, HRs with 95% CIs for PFS and OS, median PFS and OS, randomization method, analysis tool, number of patients randomized, demographic and clinical data (e.g., age, sex, ethnicity, histology), and toxicity (grade 3/4). Any disagreements were resolved by consensus, if necessary, by a third author. When additional information was required, the corresponding authors were contacted via email. All selected trials published as full-text articles were analyzed and classified using the Jadad score when possible. Studies with a Jadad score ≥3 were graded as high quality.

### 2.4 Statistical analysis

All meta-analyses were performed using the software Review Manager, version 5.3 (The Cochrane Collaboration). We performed meta-analyses of PFS, OS, and grade 3/4 toxicities after maintenance treatment versus observation alone and maintenance treatment versus continuous treatment. PFS was defined as the time from maintenance randomization to disease progression or death (not including the induction therapy time). To standardize the data, PFS values from several studies were adjusted to match the above definition. OS was defined as the time from maintenance randomization to death (not including the induction therapy time).

Heterogeneity between the studies was analyzed using the chi-squared test, with a test boundary value of *α* = 0.1. The fixed-effects model was first used to combine the HRs of each group. Significant heterogeneity was deemed present among the studies if the heterogeneity tests yielded a *P*-value of ≤.10 or an *I*^2^ value of >50%. In this case, we applied the random-effects.

Statement: Our meta-analysis does not address the subject's life, health, dignity, privacy, and other related issues. All analyses were based on previous published studies, thus no ethical approval or patient consent was required.

## 3 Results

### 3.1 Search results

**Figure 1:**
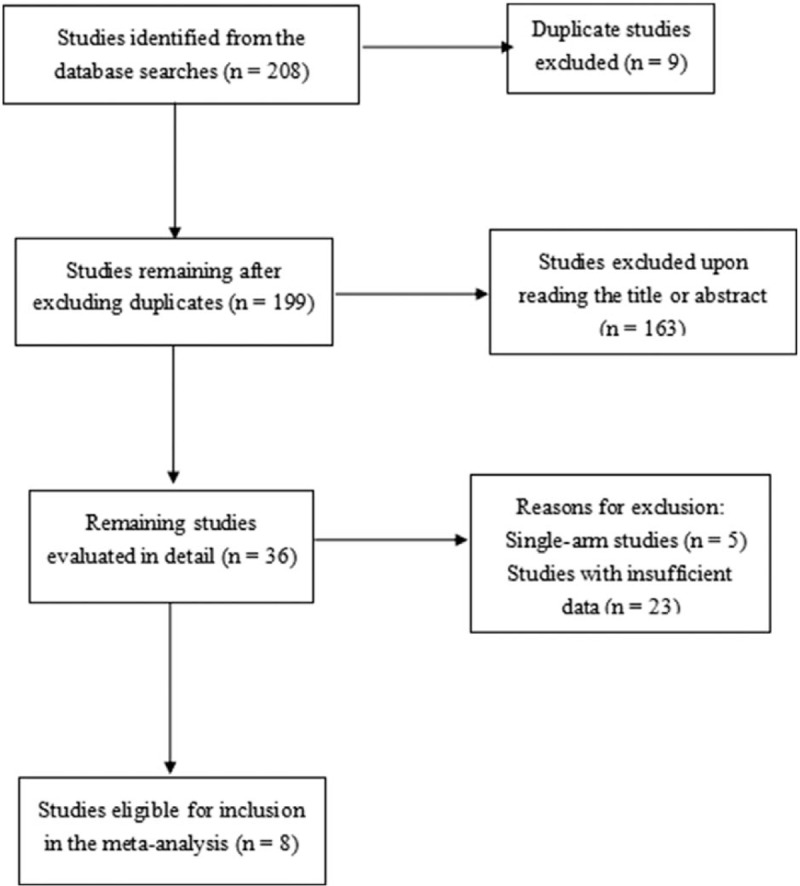
Flow chart of trial selection.

A total of 208 articles were retrieved using the initial search query. After a full-text review, 9 trials (in 8 papers), with a combined study population of 3121 mCRC patients, were included in the meta-analysis (Fig. 1). Four of these trials (AIO 0207,^[14]^ CAIRO3,^[15]^ SAKK 41/06,^[16]^ and PRODIGE 9^[17]^) compared bevacizumab-based maintenance therapy until progression with observation alone, while 5 trials (MACRO TTD,^[18]^ “Stop and Go,” ^[19]^ Nordic ACT,^[20]^ AIO 0207, and DREAM OPTIMOX3^[21]^) compared bevacizumab-based maintenance therapy with continuous treatment. The AIO 0207 study had 3 treatment arms, and thus counted as 2 trials. The baseline characteristics of all 9 trials have been summarized in Table 1. Seven of the 8 studies had a Jadad score ≥3 and were graded as high quality (Table 2).

**Table 1:**
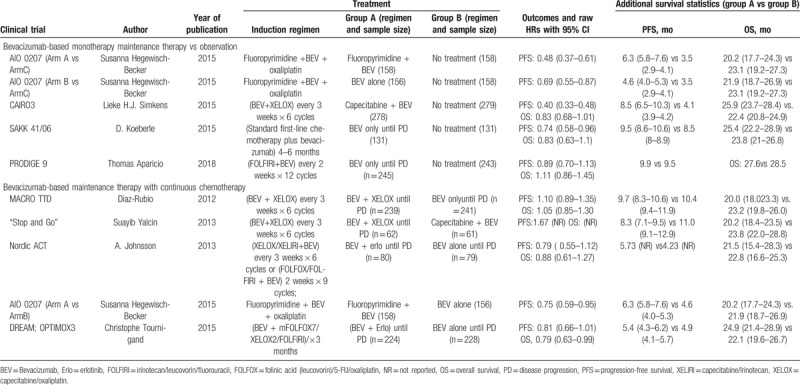
Characteristics of identified randomized controlled trials.

**Table 2:**
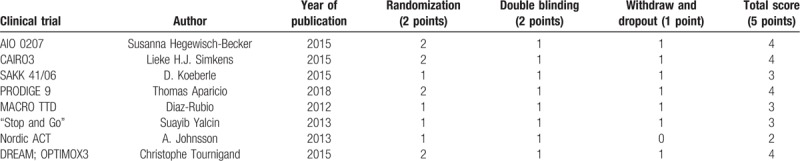
Quality of literature included in the meta-analysis (Jadad score).

### 3.2 Bevacizumab-based maintenance therapy versus observation alone

**Figure 2:**
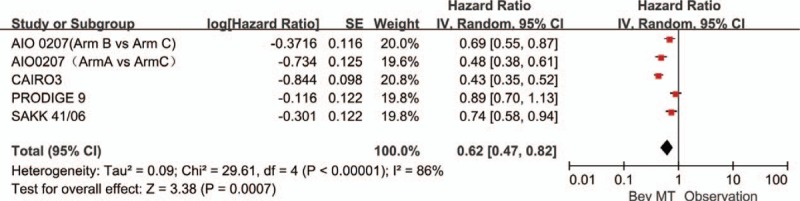
Progression-free survival in trials comparing bevacizumab-based maintenance treatment versus observation alone.

**Figure 3:**
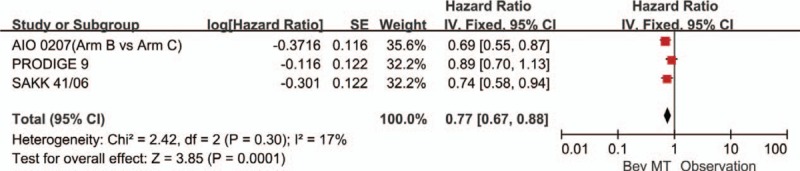
Progression-free survival in trials comparing maintenance treatment with bevacizumab monotherapy versus observation alone.

**Figure 4:**
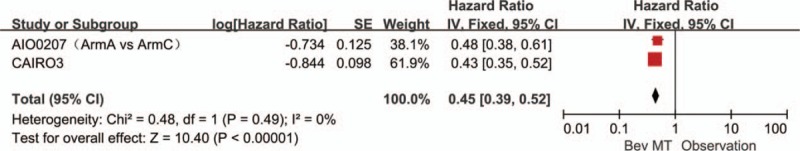
Progression-free survival in trials comparing maintenance treatment with bevacizumab plus a fluoropyrimidine versus observation alone.

**Figure 5:**
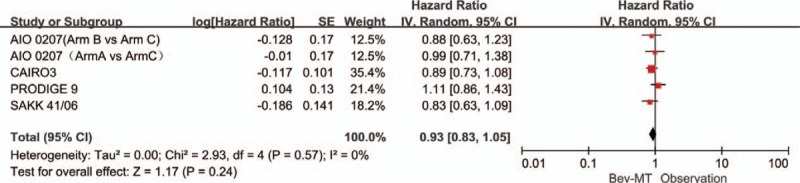
Overall survival in trials comparing bevacizumab-based maintenance treatment versus observation alone.

A total of 4 articles (5 trials) provided PFS. Significant heterogeneity was detected among these trials (*P* < .00001, *I*^2^ = 86%). Therefore, a random-effects model was used for the analysis (Fig. 2). The results showed that any bevacizumab-based maintenance therapy (with or without fluoropyrimidine) after a bevacizumab-based induction regimen improved PFS (HR = 0.62, 95% CI: 0.47–0.82). The 5 trials were separated into those studying bevacizumab monotherapy and those studying a combination of bevacizumab plus a fluoropyrimidine. The data showed that single-agent maintenance therapy with bevacizumab significantly increased PFS compared with observation alone (HR: 0.77, 95% CI: 0.67–0.88; Fig. 3). The more-intensive maintenance therapy with bevacizumab plus a fluoropyrimidine further increased PFS compared with observation alone (HR: 0.43; 95% CI: 0.35–0.52; Fig. 4). No significant difference was observed between the bevacizumab-based maintenance therapy strategies and observation alone with respect to OS (HR: 0.93, 95% CI: 0.83–1.05). In addition, no significant heterogeneity was observed in the OS analyses (*P* = 0.57, *I*^2^ = 0%; Fig. 5).

**Figure 6:**
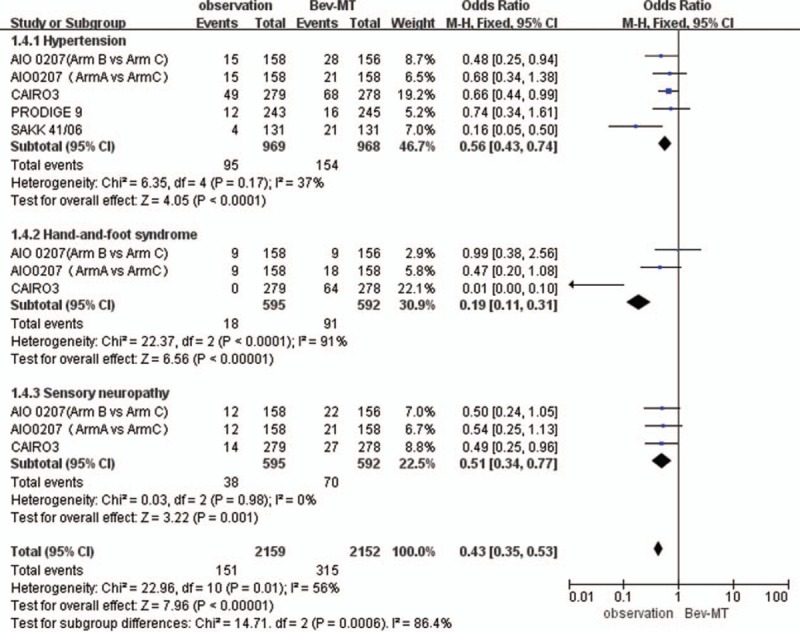
Adverse events related to bevacizumab-based maintenance treatment versus observation alone.

A subgroup analysis of toxic effects suggested that compared with the observation-alone strategy, the bevacizumab-based maintenance therapy strategies increased the incidence of hypertension (odds ratio: 0.56, 95% CI: 0.43–0.74), hand-and-foot syndrome (OR: 0.19, 95% CI: 0.11–0.31), and sensory neuropathy (OR: 0.51, 95% CI: 0.34–0.77; Fig. 6).

### 3.3 Bevacizumab-based maintenance therapy versus continuous chemotherapy

**Figure 7:**
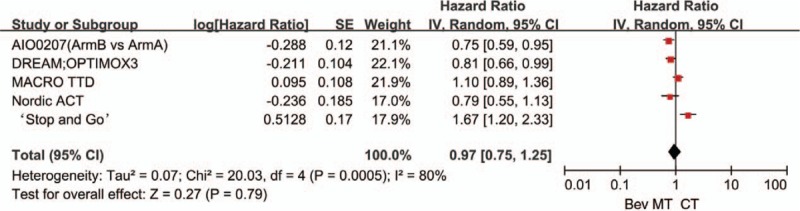
Progression-free survival in trials comparing bevacizumab-based continuous chemotherapy versus maintenance therapy.

**Figure 8:**
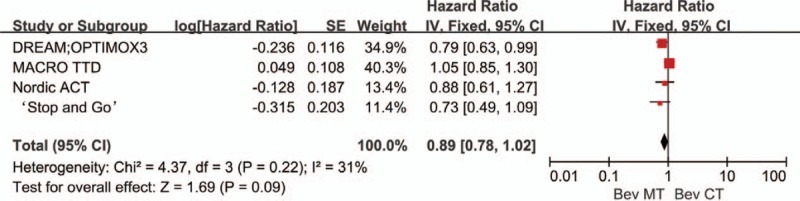
Overall survival in trials comparing bevacizumab-based continuous chemotherapy versus maintenance therapy.

Five trials comparing bevacizumab-based continuous chemotherapy (bevacizumab plus 5-FU, erlotinib, or capecitabine) with bevacizumab-based maintenance therapy provided data on PFS. Significant heterogeneity was found among these trials (*P* = .0003, *I*^2^ = 80%; Fig. 7). The data showed that compared with continuous chemotherapy, bevacizumab-based maintenance therapy (with or without fluoropyrimidine) did not significantly prolong PFS (HR: 0.91, 95% CI: 0.70–1.18). Similarly, there was no significant inter-group difference between bevacizumab-based maintenance therapy and bevacizumab-based continuous therapy with respect to OS (HR: 0.88, 95% CI: 0.74–1.04). No significant heterogeneity was observed in the OS analyses (*P* = .22, *I*^2^ = 31%; Fig. 8).

**Figure 9:**
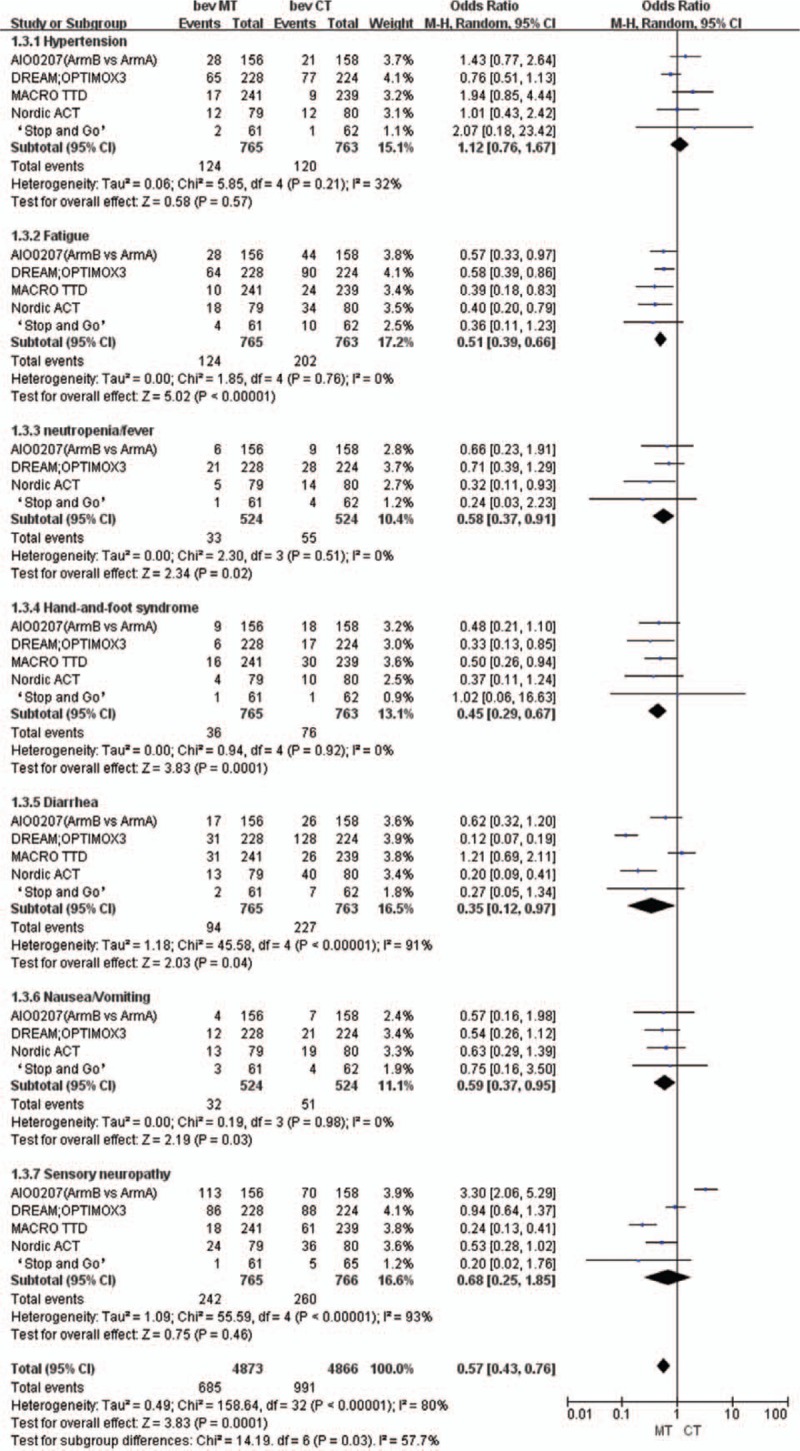
Adverse events related to bevacizumab-based maintenance therapy versus continuous chemotherapy.

Using a random-effects model, we found that compared with the continuous strategy, the maintenance strategy was associated with a lower incidence of grade 3/4 adverse events (OR: 0.57, 95% CI: 0.43–0.76; Fig. 9). The most common grade 3/4 adverse events were hypertension (OR: 1.12, 95% CI: 0.76–1.67), fatigue (OR: 0.51, 95% CI: 0.39–0.66), neutropenia/fever (OR: 0.58, 95% CI: 0.37–0.91), hand-and-foot syndrome (OR: 0.45, 95% CI: 0.29–0.67), diarrhea (OR: 0.35, 95% CI: 0.12–0.97), nausea/vomiting (OR: 0.59, 95% CI: 0.37–0.95), and sensory neuropathy (OR: 0.68, 95% CI: 0.25–1.85).

### 3.4 Publication bias

**Figure 10:**
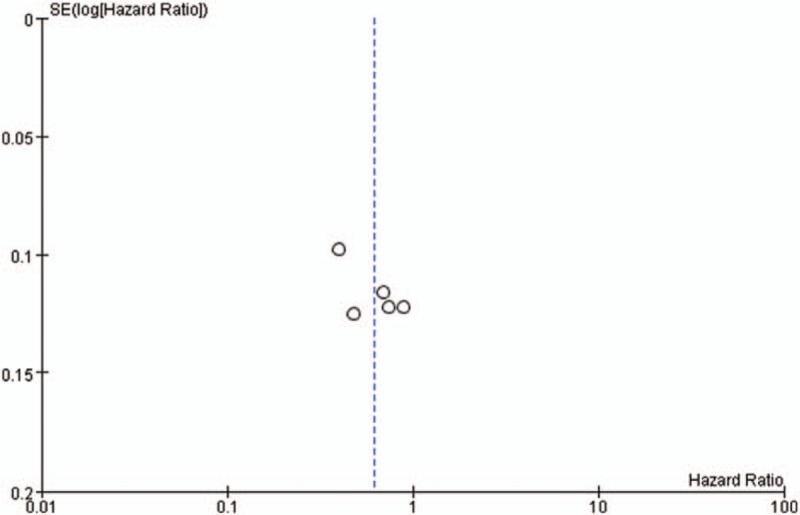
Publication bias in trials comparing bevacizumab-based maintenance therapy versus observation alone.

**Figure 11:**
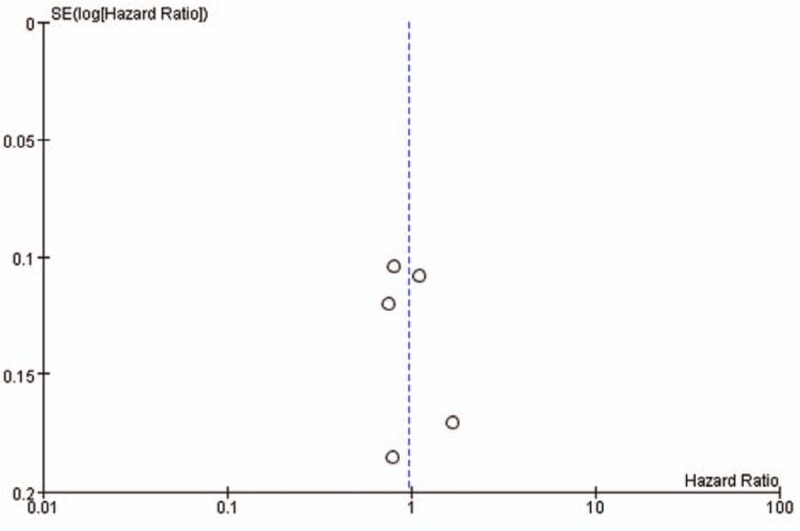
Publication bias in trials comparing bevacizumab-based maintenance therapy versus continuous chemotherapy maintenance therapy versus observation alone.

We generated funnel plots of PFS indicators in both comparisons (Figs. 10 and 11). The inverted funnel plots were symmetric, indicating that there was no publication bias among the included studies.

## 4 Discussion

Bevacizumab, a humanized monoclonal antibody against VEGF, selectively blocks VEGF binding to the VEGFR-1 and VEGFR-2 receptors, thereby inhibiting the tumor angiogenesis.^[22]^ The addition of bevacizumab to 5-FU-based combination chemotherapy results in improvements in the overall response rate, PFS, and OS among mCRC patients.^[10]^ This meta-analysis has clearly showed that compared with observation alone, bevacizumab-based maintenance therapy has a significant benefit in terms of PFS and has a trend toward prolonging OS in mCRC patients who benefit from first-line therapy. Compared with observation alone, bevacizumab-based maintenance therapy significantly improved PFS (HR: 0.62, 95% CI: 0.47–0.82) and showed a trend toward prolonged OS (HR: 0.93, 95% CI: 0.83–1.05). Although the toxicity of bevacizumab-based maintenance therapy was increased, the patients were well tolerated. However, there were no difference of PFS (HR: 0.91, 95% CI: 0.70–1.18) and OS (HR: 0.88, 95% CI: 0.74–1.04) between the bevacizumab alone and combination chemotherapy groups. Furthermore, the toxicity including diarrhea and sensory neuropathy was increased in bevacizumab combination chemotherapy group. Together, these data suggested the bevacizumab alone maintenance therapy maybe the optimal strategy for mCRCs patients.

Although the OS have not significantly difference between maintenance therapy with bevacizumab alone versus observation alone, there were significant benefits in terms of PFS. However, different follow-up intervals and assessment methods have an impact on PFS data. In the AIO 0207 trial, the median PFS after first-line therapy was slightly but significantly improved by bevacizumab maintenance compared with observation (4.6 vs 3.5 months). Non-inferiority for bevacizumab alone was demonstrated for the primary endpoint in AIO 0207, and during the maintenance phases, CT or MRI scans were done every 6 weeks. In the SAKK 41/06 trial, the median PFS was 4.1 months for the bevacizumab maintenance therapy arm versus 2.9 months for the observation alone arm. The follow-up time and equipment were inconsistent in each trial. Non-inferiority could not be demonstrated for continuing bevacizumab monotherapy, CT scans were also done every 6 weeks until disease progression in SAKK 41/06 trials. CT scans were done every 8 weeks to assess the disease status in the PRODIGE 9 trial. There may be a small difference between trials from the time to start maintenance treatment to the first progression between patients treated with bevacizumab and those in the observation group.

Cost-effectiveness was mentioned in the CAIRO3 trial^[23]^ and the SAKK41/06 trial. In the CAIRO3 experiment, bevacizumab-based maintenance therapy (CAP-B) cost on average €36,845 more than the observation-alone strategy. In the SAKK41 trial, the average cost (in US dollars) per patient was $37,596 (range, $4794–$229,038) for the bevacizumab maintenance arm and $8180 (range, $330–$83,465) for the observation group. Compared with observation, maintenance therapy leads to an improvement in the quality of life, but it also leads to an increase in costs. Although there is no consensus on the cost-effectiveness thresholds for cancer treatment, the cost-effectiveness of maintenance treatment cannot be ignored.

The first-line treatment should also be considered as a potential source of bias. Trials of oxaliplatin-based first-line therapy (100% of patients in the CAIRO3 and AIO KRK 0207 trials and 62% of patients in the SAKK 41/06 trial with 31% also receiving irinotecan) that compared maintenance therapy with observation alone showed that maintenance treatment had no significant effect to extend OS. In the PRODIGE 9 trial (100% of patients receiving irinotecan), the irinotecan-based combination with bevacizumab maintenance therapy resulted in prolong the OS. The use of oxaliplatin has cumulative toxicity, especially neurotoxicity. Using irinotecan-based chemotherapy may be more feasible than oxaliplatin-based chemotherapy, which may require more clinical trial on maintenance therapy to further confirm.

CRCs can be characterized by their primary tumor location within the colon. The right and left sides of the colon differ in the clinical features, and chromosomal and molecular characteristics. For the above reasons, in a large number of clinical studies on mCRC patients, the location of the primary colon tumor also has an effect on the therapeutic response.^[24]^ Based on mature survival data from AIO 0207 trial, patients with left-sided tumors showed a median OS of 24.8 months compared with the right-sided cohort with 18.4 months. In a multivariable model, location of the primary tumor proved to be an prognostic factor.^[25]^ In the AIO 0207 and PRODIGE 9 trials, World Health Organization status ≥2, and more than one metastatic site were associated with a shorter OS. Tumor BRAF mutations were a poor prognostic factor in both trials. In addition, in the CAIRO3 and PRODIGE 9 trials, patients with synchronized metastases who were given bevacizumab-based maintenance therapy had a better OS than the observation-alone group. The PRODIGE 9 trial recommended that patients with poor prognostic molecular markers were unsuitable for maintenance therapy strategies. However, the OPTIMOX series of studies^[26]^ suggested that high-risk patients with poor prognosis can receive maintenance therapy, while observation after first-line therapy may be a more rational strategy in low-risk patients with a good prognosis.

While drawing clear recommendations for optimal maintenance treatment options, we were able to identify key differences in PFS in clinical trials comparing single bevacizumab maintenance therapy with combination chemotherapy. The results of the MACRO trial suggest that single-agent maintenance therapy with bevacizumab may be an appropriate choice for mCRC patients. The “Stop and Go” trial proposes that maintenance therapy with bevacizumab plus capecitabine after first-line chemotherapy with 6 cycles of bevacizumab + XELOX can be considered an appropriate choice. In the Nordic ACT trial and OPTIMOX3 trial, after first-line chemotherapy, maintenance therapy with a combination of erlotinib and bevacizumab demonstrated better PFS than did maintenance therapy with bevacizumab alone. The former is a new non-chemotherapy-based maintenance regimen, whose relatively modest efficacy seems to be outweighed by its significant toxicity, especially rash, diarrhea, and fatigue. Ongoing clinical and translational studies focus on identifying subgroups of patients that may benefit from erlotinib in the maintenance setting. The AIO 0207 trial investigated whether observation strategy or bevacizumab alone are non-inferior to a fluoropyrimidine plus bevacizumab, following first-line treatment with a fluoropyrimidine plus oxaliplatin plus bevacizumab. The results showed that maintenance with a fluoropyrimidine plus bevacizumab provided longer PFS than did de-escalation to bevacizumab monotherapy or to no treatment at all. Furthermore, we found that the incidence of adverse events tended to be higher after the bevacizumab combination first-line treatment regiments than after bevacizumab maintenance treatment or observation alone. Although the 8 trials showed that compared with observation alone, bevacizumab-based maintenance significantly prolonged PFS and improved quality of Life, but without improve OS. Further stratification based on the risk factors, such as primary site of colon cancer, BRAF V600 and RAS mutation status, physical status and number of metastatic sites and so on, the clinical trials of maintenance therapy based on further stratification may be prolong the patient's OS while increasing PFS.

The current meta-analysis has several limitations. First of all, this is a meta-analysis at study level. We could not obtain individual patient data from the publication, thus we could not incorporate patients variables into the analysis. Second, there were heterogeneities in the trial design (superiority in CAIRO3 and noninferiority in AIO, KRK 0207, and SAKK 41/06). Furthermore, the variability in the baseline patient characteristics (e.g., the trial design, differences in induction treatments and fluoropyrimidine maintenance schedules, induction treatment duration, and drug intensity) could not be controlled for. This necessitated adjusting the data according to the study design, which should be considered as a potential source of bias.

## 5 Conclusion

Compared with observation alone, bevacizumab-based maintenance therapy significantly prolonged the PFS of mCRC patients. Bevacizumab-based maintenance therapy seems have comparable effectiveness (in terms of PFS and OS) to single drug maintenance chemotherapy with lower cumulative grade 3/4 toxicity. Thus, maintenance therapy with bevacizumab may be a valid option for mCRC patients. Although maintenance therapy has demonstrated significant benefits in clinical studies, the treatment should still be individualized. Irinotecan-based first-line chemotherapy may be more likely to prolong OS than oxaliplatin-based induction chemotherapy, more clinical studies are needed to confirm. Clinical studies are conducted on the basis of further stratification, which may prolong the OS of patients with mCRC.

## Author contributions

**Funding acquisition:** Hongbo Ma.

**Investigation:** Hongbo Ma, Nan Tang, Yanyan Li.

**Methodology:** Miaomiao Tao, Xianquan Zhang.

**Project administration:** Hongbo Ma, Qi Zhou.

**Resources:** Hongbo Ma, Yanyan Li.

**Software:** Hongbo Ma, Xiaoli Wu, Miaomiao Tao.

**Writing – original draft:** Hongbo Ma.

**Writing – review & editing:** Hongbo Ma, Xianquan Zhang, Qi Zhou.

Qi Zhou orcid: 0000-0002-6391-0453

Hongbo Ma orcid: 0000-0003-0712-9926.
